# Biochemical and Molecular Characterization of Potential Phosphate-Solubilizing Bacteria in Acid Sulfate Soils and Their Beneficial Effects on Rice Growth

**DOI:** 10.1371/journal.pone.0097241

**Published:** 2014-10-06

**Authors:** Qurban Ali Panhwar, Umme Aminun Naher, Shamshuddin Jusop, Radziah Othman, Md Abdul Latif, Mohd Razi Ismail

**Affiliations:** 1 Department of Land Management, Faculty of Agriculture, Universiti Putra Malaysia (UPM), Serdang, Selangor, Malaysia; 2 Institute of Tropical Agriculture, Universiti Putra Malaysia (UPM), Serdang, Selangor, Malaysia; 3 Bangladesh Rice Research Institute, Gazipur, Bangladesh; Centro de Investigación y de Estudios Avanzados del IPN, Mexico

## Abstract

A study was conducted to determine the total microbial population, the occurrence of growth promoting bacteria and their beneficial traits in acid sulfate soils. The mechanisms by which the bacteria enhance rice seedlings grown under high Al and low pH stress were investigated. Soils and rice root samples were randomly collected from four sites in the study area (Kelantan, Malaysia). The topsoil pH and exchangeable Al ranged from 3.3 to 4.7 and 1.24 to 4.25 cmol_c_ kg^−1^, respectively, which are considered unsuitable for rice production. Total bacterial and actinomycetes population in the acidic soils were found to be higher than fungal populations. A total of 21 phosphate-solubilizing bacteria (PSB) including 19 N_2_-fixing strains were isolated from the acid sulfate soil. Using 16S rRNA gene sequence analysis, three potential PSB strains based on their beneficial characteristics were identified (*Burkholderia thailandensis*, *Sphingomonas pituitosa* and *Burkholderia seminalis*). The isolated strains were capable of producing indoleacetic acid (IAA) and organic acids that were able to reduce Al availability via a chelation process. These PSB isolates solubilized P (43.65%) existing in the growth media within 72 hours of incubation. Seedling of rice variety, MR 219, grown at pH 4, and with different concentrations of Al (0, 50 and 100 µM) was inoculated with these PSB strains. Results showed that the bacteria increased the pH with a concomitant reduction in Al concentration, which translated into better rice growth. The improved root volume and seedling dry weight of the inoculated plants indicated the potential of these isolates to be used in a bio-fertilizer formulation for rice cultivation on acid sulfate soils.

## Introduction

Acid sulfate soils are sporadically spread throughout the coastal plains around the globe. The estimated worldwide extent of acid sulfate soils is about 24 million hectares [1]. In Malaysia, acid sulfate soils mainly occur in the west coastal plains of Peninsular Malaysia and Sarawak (Borneo Island). The soils are characterized by the presence of pyrite (FeS_2_), which is associated with high acidity and Al content, resulting from its oxidation upon exposure to the atmosphere [2]. Under acidic soil conditions, Al^3+^ restricts the growth of roots either by inhibiting cell division, cell elongation or both [3]. It is also known that rice grown on acid sulfate soils are subjected to Fe^2+^ toxicity [4].

Acid sulfate soils are normally not suitable for crop production unless they are properly ameliorated and their fertility improved [5]. They usually contain high concentration of Al and Fe which influence the biochemical properties of the soils. There are only a few acid-tolerant plant species and microbes that are able to survive in these soils. The soils contain low amount of total microorganisms, which vary considerably depending on the vegetation type and soil management. Microorganisms perform a major role in nutritional chains that are an important part of the biological balance in soils [6]. Bacteria are important for closing nutrient and geochemical cycles, involving carbon, nitrogen, sulfur and phosphorous. Beside chemical treatment, addition of microbes may improve nutrient availability in soils (especially phosphorus) and reduce Al toxicity.

There are some species of bacteria which have the potential to mineralize and solubilize organic and inorganic phosphorus in soil [7]. Bacterial strains such as *Pseudomonas*, *Bacillus* and *Rhizobium* are the dominant phosphate solubilizers [8] and single novel genus *Sphingomonas* spp. has the capability to fix nitrogen [9]. Moreover, in recent years, a growing number of *Burkholderia* strains and species have also been reported as plant-associated bacteria. *Burkholderia* spp. can be free-living in the rhizosphere as well as epiphytic or endophytic, obligate endosymbionts or phytopathogens [10]. Among the beneficial properties of these bacteria is the production of plant growth promoting phytohormone, polysaccharides and organic acids that are important for rice growth. Indoleacetic acid produced by the bacteria is known to promote an extensive root architecture capable of absorbing nutrient elements efficiently from the surroundings which ultimately improves rice growth [11]–[12].

PSB produce large amounts of organic acids [13] resulting in Al binding via a chelation process which is a plausible mechanism for reducing Al toxicity to rice roots in acid sulfate soils. Few attempts have been made to isolate and characterize these potential microorganisms living in the rhizosphere of rice plants grown in acid sulfate soils. Hence, the present study was undertaken to isolate and enumerate microbial occurrences in acid sulfate soils, and to identify beneficial isolates such as N_2_-fixing and P- solubilizing bacteria that have important role in nutrient cycling, reducing Al toxicity and producing IAA in an acid sulfate soil environment.

## Materials and Methods

### Sampling location and isolation of microbes

#### Experimental site and conditions

This experiment was conducted at the Soil Microbiology Laboratory, Department of Land Management, Faculty of Agriculture, Universiti Putra Malaysia, Serdang, Malaysia. Soil samples were randomly collected from four sites in an acid sulfate soil area at Semerak, Kelantan, Malaysia which is located at a latitude of 30.01°N and longitude of 101°.70E.

We are working in a Government agency (University) so there is no need to take any permission for research activities from any one as we are working in a Government Project” Long term Research Grant Scheme” (LRGS) by the Ministry of Higher Education Malaysia. Hence, no specific permissions were required for these locations/activities. It has no any conflict on this issue.” The methods to determine the soil physico-chemical analyses are given in Table 1.

**Table 1:** Analytical methods used in the study.

S No.	Analysis	Procedure
1	Soil pH and EC	Soil: water (1∶2.5) extract using PHM210 standard pH meter and EC meter by glass electrode [14]
2	CEC	1 M NH_4_OAc solution buffered at pH 7.0 was used [14]
3	Soil texture	Pipette method [15]
4	Total N	Kjeldahl digestion method [16]
5	Exchangeable cations (Ca, Mg, K)	Extracted by 1M NH_4_OAc solution at pH 7 [14]
6	Exchangeable Al	Extracted by 1 M KCl solution
7	Total carbon	CN analyzer (LECO CR-412)
8	Micronutrients (Cu, Mn, Zn, Fe)	Inductivity coupled plasma - atomic emission spectroscopy (ICP-AES)

ICP-AES = inductively coupled plasma atomic emission spectroscopy, CEC = cation exchange capacity.

#### Enumeration of the total microbial population from rice cultivated on acid sulfate soil

A series of 10-fold dilutions were prepared up to 10^−8^ for soil and rhizosphere microbial population determinations using the spread plate count method. Total fungal, bacterial and actinomycetes populations were determined using potato dextrose agar (PDA), nutrient agar (NA), and actinomycetes agar plates, respectively in five replicates.

#### Enumeration of PSB from rice cultivated on acid sulfate soil

Phosphate-solubilizing bacterial (PSB) population was determined from the soil, rhizosphere and endosphere using selective media plates at various pH levels: (i) the National Botanical Research Institute's phosphate growth medium (NBRIP) [17] at pH 5.0 and 6.7; and (ii) PDYA-AlPO_4_ at pH 3.5 and 5. For the determination of the rhizosphere population, approximately 3 g of rice plant roots with its adhering soil were transferred into conical flask containing 99 mL of sterile distilled water and the contents were vigorously shaken. A 10-fold dilution series was prepared up to 10^−8^ and 0.1 mL aliquots were spread on selective media and incubated at 28±2°C in an incubator. For the determination of the endophytic population, fresh roots were taken and surface sterilized with 70% ethanol for 5 minutes and treated with 3% Clorox for 30 seconds [11]. Roots were cut into small pieces and surface sterilized by dipping into 95% ethanol and a flame. Surface sterilized roots were homogenized using a sterilized mortar and pestle. Endophytic PSB populations were determined using the total plate count method in five replicates.

#### Estimation of diazotrophs from rice cultivated on acid sulfate soil

A series of 10-fold dilutions were prepared up to 10^−8^ using rhizosphere and non-rhizosphere soil and the diazotroph populations were determined using the most-probable number (MPN) method in nitrogen free (Nfb) semi-solid medium in five replicates. The Nfb semi-solid medium [18] contained 5 g malic acid, 0.5 g K_2_HPO_4_, 0.2 g MgSO_4_. 7 H_2_O, 0.1 g NaCl, 0.02 g CaCl_2_ and 0.5% bromothymol blue in 0.2 N KOH (2 mL), 1.64% Fe-EDTA solution (4 mL) and 2 g agar.

### Determination of the beneficial traits of isolated bacteria

#### Determination of indoleacetic acid (IAA)

The isolates were inoculated in nutrient agar (NB) broth with the addition of tryptophan (2 mg L^−1^) and incubated at 28±2°C for 48 hours. The culture was centrifuged at 7000 rpm for 7 minutes and 1.0 mL of the supernatant was mixed with 2 mL of Salkowsky's reagent [19]. The IAA concentration was determined using a spectrophotometer at 535 µm in five replicates.

### Phosphate solubilization

#### Phosphate solubilization on media plates

The phosphate solubilizing activities of the isolates were assayed by spotting 10 µL of the cultures on different phosphate containing media such as NBRIP [17], Pikovaskaya, Christmas Island Rock Phosphate (CIRP) and PDYA-AlP in five replicates. The plates were incubated at 30°C for one week and the phosphate solubilization efficiency was measured [20]:
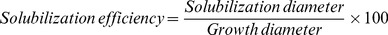
(1)


#### Phosphate solubilization in broth culture

The isolated PSB strains were evaluated for their phosphate-solubilizing activity in broth culture. Three different media containing insoluble phosphate: i) calcium phosphate in NBRIP broth [17] containing (g L^−1^) MgCl_2_.6H_2_O_5_ g, MgSO_4_.H_2_O 0.25 g, KCl 0.2 g, (NH_4_)_2_SO_4_ 0.1 g, Ca_3_(PO_4_)_2_ amended with glucose 10 g; ii) CIRP broth modified from NBRIP supplemented with phosphate rock (CIRP) instead of calcium phosphate; and iii) PDYA-AlP broth [21] containing (g L^1^): PDA agar 39 g, yeast extract 2 g, sterilized 10% K_2_HPO_4_, and 10% AlCl_3_ (100 mL). Each media (200 mL) were inoculated with PSB inoculum and were incubated at 30°C on a Kottermann 4020 shaker at 80 rpm for 3 days. Each treatment was replicated five times.

#### Determination of the population in broth culture

One milliliter of broth was taken from the respective flasks at different periods (initial & after 72 h) for determination of the bacterial population. A series of 10-fold dilutions were prepared up to 10^−10^. The population was determined using the drop plate count method according to the method of Somasegaran and Hoben [22].

#### Determination of phosphorus solubilization in broth cultures

Exactly 2 mL of samples were taken for P determination. The samples were first allowed to sediment for 15 minutes and were then centrifuged at 4000× g for 5 minutes. The supernatant was filtered through 0.2 µm filter paper and kept at −20°C until analysis. The available P was determined according to the published methods [23].

#### Nitrogen-fixing activities

The N_2_-fixing activity was determined using the Nfb semi-solid liquid medium [18]. The presence of pellicle formation below the surface of media indicated the N_2_-fixing activity.

### Molecular identification of PSB strains

#### DNA extraction and primers

The identification of PSB was carried out on the basis of 16S rRNA gene sequencing. The genomic DNA of PSB isolates was extracted by the Qiagen DNeasy Plant Mini Kit (Qiagen, Valencia, CA). Forward primers D1 (5-AGAGTTTGATCCTGGCTCAG-3) and reverse P2 (3-ACGGCTACCTTGTTACGACTT-5) were used for amplification of 16S rRNA gene [24]. Each sample was replicated five times.

#### PCR protocols and gel electrophoresis

The total PCR reaction mixture was 50.0 µL, comprising 200 µM dNTPs, 50 µM of each primer, 1× PCR buffer, 3 U *Taq* polymerase and 100 µg genomic DNA. The thermal cycler (MJ Mini personal Thermal Cycler, Bio-Rad, Model- PTC-1148) conditions were as follows: 95°C for 3 minutes, followed by 30 cycles of denaturation at 95°C for 1 minute, annealing at 48°C for 1 minute and primer extension at 72°C for 2 minutes. This was followed by a final extension at 72°C for 10 minutes. The reaction products were separated by running 5 µL of the PCR reaction mixture in a 1.0% (w/v) agarose gel and staining the bands with ethidium bromide.

#### Strain identification using gene sequencing

Three potential isolates with greater beneficial characteristics were selected for 16S rRNA gene sequencing analysis. Sequence data were aligned and compared with the available standard sequences of bacterial lineage in the National Center for Biotechnology Information GenBank (http://www.ncbi.nlm.nih.gov/) using BLAST [25]. A phylogenetic tree was constructed by the neighbor-joining method using the software MEGA 4 [26]. The obtained sequences were deposited in the European Molecular Biology Laboratory data (accession number NR 074312.1, NR 042635.1 and NR 25363.1).

#### Principal Components Analysis

Principal Components Analysis (PCA) is an ordination technique. Here we performed an eigen analysis of the covariance matrix. The eigen value is a measure of the strength of an axis, the amount of variation along an axis, and ideally the importance of an ecological gradient. The precise meaning depends on the ordination method used. Two- dimensional graph (two axes of PCA) was constructed using co-variance matrix and find out the variation among the characters of 21 bacterial isolates.

#### Efficiency of isolates to improve rice seedling growth with high Al and low pH

Three potential phosphate-solubilizing bacterial strains (PSB7, PSB17 and PSB21) were selected on the basis of their beneficial characteristics. Seven-days-old MR 219 rice seedlings were grown in Hoagland solution containing different concentrations of Al (0, 50 and 100 µM) with five replicates. The initial pH of the solution was adjusted to 4.0. Rice seedlings were harvested 21 days after sowing. The bacterial population, plant dry biomass, solution pH and organic acids were determined soon thereafter.

#### Determination of the microbial population at different Al concentrations

One milliliter of broth was taken from the respective flasks at different time periods (6, 12, 24, and 48 h) for the determination of bacterial growth. A series of 10-fold dilution were prepared up to 10^−10^. The population was determined using the drop plate count method [22].

#### Determination of organic acids

About 20 µL of the samples from each treatment were injected into HPLC with a UV detector set at 210 nm. A Rezex ROA-organic acid “H^+^” (8%) column (250×4.6 mm) from Phenomenex Co. was used, the mobile phase was 0.005 N H_2_SO_4_ with a flow rate of 0.17 mL min^−1^.

#### Determination of root morphology

The root morphology of the rice plants was determined using a root scanner (model Epson Expression 1680 equipped with root scanning analysis software). Total root length (cm), total surface area (cm^2^) and total volume (cm^3^) were quantified using a scanner (Expression 1680, Epson) equipped with a 2 cm deep plexiglass tank (20.30 cm) filled with up H_2_O [27]. The scanner was connected to a computer and scanned data were processed by Win-Rhizo software (Regent Instruments Inc., Québec, Canada).

### Statistical analysis

All data were statistically analyzed using SAS Software (Version 9.2), and treatment means were separated using Tukey's test (P<0.05).

## Results

### Properties of the acid sulfate soils

The pH of the acid sulfate soils under study ranged from 3.3 to 4.7 pH with values decreasing with depth (Table 2). The soils were taxonomically classified as Typic Sulfaquepts. Soils of this nature normally contain pyrite in the subsoil, having pH<3.5 below a depth of 50 cm [2]. It is the oxidation of this pyrite that produces acidity and the subsequent release of Al and/or Fe into the environment. The low soil pH is consistent with the presence of high exchangeable Al in the soils. Exchangeable Al in the topsoil at all sampling sites ranged from 1.24 to 4.25 cmol_c_ kg^−1^, occurring at a toxic level for rice growth. Total N and exchangeable K were found to be at sufficient levels for rice growth, while available P, exchangeable Ca and Mg were insufficient. The Zn, Cu and Mn contents were low, while extractable Fe was high (124 to 181 mg kg^−1^) (Table 2). Total C was high in the topsoil with values above 2% and it decreased consistently with depth. The high organic matter in the topsoil would have a profound effect on the availability of Al and Fe, which can be partly fixed (chelated) by it and hence deactivated. Chelated Al and Fe are non-toxic to rice plants in the field.

**Table 2:** Chemical characteristics of the acid sulfate soils.

Site	Soil depth	Soil pH	Total C	Total N	Avail. P	K	Al	Ca	Mg	Fe	Zn	Cu	Mn
						-------------------- Exchangeable -------------------				------------ Extractable -------------			
	(cm)		---------- (%) -----------		(mg kg^−1^)	---------------- (cmol_c_ kg^−1^) ----------------				------------- (mg kg^−1^) -------------			
1	0–15	4.0b	2.1c	0.18b	26.3a	0.05b	1.7cd	0.43c	1.0bc	174abc	1.6ab	3.2a	8.1ab
	15–30	4.0b	1.6d	0.14c	19.1b	0.05b	2.1c	0.40c	0.9cd	170abc	1.6ab	2.5b	7.8ab
	30–45	3.8bc	1.2e	0.10d	13.1def	0.04c	4.5b	0.30d	0.6ef	129bcd	0.9d	2.3bc	7.4abc
	45–60	3.6c	0.9f	0.09d	12.9def	0.04c	5.5a	0.25d	0.5ef	124d	0.7d	2.0bcd	6.4bcd
2	0–15	4.7a	2.9a	0.17b	25.2a	0.06a	1.24d	0.57b	0.7de	181a	2.3a	2.4b	8.8a
	15–30	3.6c	1.1e	0.12c	16.6bc	0.04c	1.5cd	0.43c	0.5ef	176ab	1.8a	1.9cde	8.2ab
	30–45	3.4de	1.1e	0.09de	15.5cd	0.04c	1.7cd	0.40c	0.4f	167abcd	1.8a	1.7def	7.9ab
	45–60	3.3e	0.9f	0.08de	11.4f	0.03d	1.9c	0.12e	0.4f	163bcd	1.5b	1.5ef	6.0bcd
3	0–15	3.8bc	2.3b	0.21a	19.2b	0.05b	1.8cd	0.80a	1.3a	180a	2.0a	1.4ef	7.5ab
	15–30	3.5d	1.1e	0.13c	14.8cde	0.04c	1.9c	0.73a	1.2b	178a	1.3bc	1.5def	6.1cd
	30–45	3.4de	0.9f	0.09d	12.2ef	0.04c	1.9c	0.63b	0.94bc	145cd	1.2c	1.5ef	5.3cd
	45–60	3.3e	0.6g	0.06e	11.6f	0.04c	4.3b	0.60b	0.7de	124d	1.1c	1.1f	4.5d
4	0–15	3.8bc	2.2b	0.20a	19.1b	0.05b	1.7cd	0.72a	1.2b	179a	1.97a	1.3ef	7.2ab
	15–30	3.6c	1.15e	0.11c	14.3cde	0.04c	1.8cd	0.70a	1.0bc	170abc	1.2c	1.4ef	6.0cd
	30–45	3.5d	1.01ef	0.08de	11.5f	0.03d	2.03c	0.60b	0.94bc	154c	1.1c	1.4ef	5.2cd
	45–60	3.4de	0.5g	0.05e	10.7fg	0.03d	4.03b	0.56b	0.8de	130d	1.0c	1.2f	4.3d

Data values are means of five replicates. Means followed by the same letter within a column are not significantly different (P<0.05).

### Total population of microorganisms in the rhizosphere, non-rhizosphere and endosphere

Higher microbial populations were found in the rice rhizosphere compared to the non-rhizosphere. The total bacterial and actinomycetes population were higher than fungal populations (Table 3) and higher PSB populations were observed on NBRIP plates compared to PDYA-AlP media plates (Table 4). However, there were no significant population differences of PSB present in the NBRIP media plates at media pH of 5.0 and 6.7. The highest diazotrophic population was found in the endosphere (25×10^6^ cfu root g^−1^) and the lowest was in the non-rhizosphere (13×10^3^ cfu soil g^−1^) (Table 4).

**Table 3:** Total microbial population of microorganisms isolated from the acid sulfate soils.

Site	Bacterial		Fungal		Actinomycetes	
	------------------------------------- (*cfu soil g^−1^) ------------------------------------					
	Rhizosphere	Non-rhizosphere	Rhizosphere	Non-rhizosphere	Rhizosphere	Non-rhizosphere
1	1.5×10^7b^	7.1×10^5a^	7.0×10^3ab^	6.0×10^3ab^	3.0×10^4c^	2.1×10^5a^
2	9.0×10^6c^	3.5×10^5b^	10.0×10^3ab^	3.0×10^3b^	8.0×10^4b^	3.0×10^4c^
3	2.8×10^7a^	1.8×10^5c^	7.0×10^3a^	9.0×10^3a^	1.2×10^5ab^	10.0×10^4ab^
4	1.9×10^7ab^	1.5×10^5c^	2.0×10^3b^	5.0×10^3ab^	1.2×10^5ab^	4.0×10^4c^

*cfu = colony forming unit, Data values are means of five replicates.

Means followed by the same letter within a column are not significantly different (P<0.05).

**Table 4:** Phosphate-solubilizing bacteria and diazotroph population from rice cultivated on acid sulfate soils.

Site	Phosphate-solubilizing bacterial population												Diazotrophs population		
	Rhizosphere				Non-rhizosphere				Endosphere				Rhizosphere	Non-rhizosphere	Endosphere
	(*cfu soil g^−1^)				(cfu soil g^−1^)				(cfu root g^−1^)				(cfu soil g^−1^)	(cfu soil g^−1^)	(cfu root g^−1^)
	†NBRIP		‡PDYA-AlP		NBRIP		PDYA-AlP		NBRIP		PDYA-AlP		------------ N-free media ------------		
	pH 5.0	pH 6.7	pH 3.5	pH 5.0	pH 5.0	pH 6.7	pH 3.5	pH 5.0	pH 5.0	pH 6.7	pH 3.5	pH 5.0	23×10^5b^	24×10^3b^	25×10^6a^
1	2×10^7a^	4×10^4b^	-	-	5.7×10^4a^	3×10^4a^	-	-	28×10^4a^	19×10^4b^	-	-	15×10^6a^	3×10^4a^	11×10^5b^
2	70×10^4b^	41×10^4b^	-	-	2×10^3b^	3×10^3b^	-	-	8×10^4^	4×10^4a^	-	-	16×10^5b^	13×10^3b^	38×10^5b^
3	57×10^4b^	60×10^4b^	-	5×10^4a^	3×10^4a^	21×10^4a^	-	6×10^4a^	9×10^4^	9×10^4a^	-	-	18×10^6a^	27×10^3b^	3×10^6a^
4	24×10^4b^	5×10^4b^	-	2×10^4a^	2×10^4a^	21×10^4a^	-	5×10^4a^	50×10^4a^	71×10^4a^	-	5×10^4a^	23×10^5b^	24×10^3b^	25×10^6a^

*cfu = colony forming unit,

† NBRIP = National Botanical Research Institute's phosphate growth medium,

‡ PDYA-AlP = Potato dextrose Yeast Agar- Aluminium Phosphate respectively.

Data values are means of five replicates. Means followed by the same letter within a column are not significantly different (P<0.05).

### Biochemical properties of the strains isolated from rice cultivated on acid sulfate soils

The indigenous bacterial isolates were capable of producing indoleacetic acid (IAA). Among all the isolates, PSB21 (14.96 mg L^−1^), followed by PSB7 (13.16 mg L^−1^) produced high IAA levels as compared to the other isolates. The lowest amount of IAA was produced by PSB20 (Table 5). A total of 21 phosphate-solubilizing bacterial (PSB) isolates were identified from the acid sulfate soils. However, only 19 strains were able to grow in nitrogen-free medium (Table 5).

**Table 5:** Biochemical properties of the isolated PSB strains.

Isolates	IAA production (mg L^−1^)	Nfb activity	P solubilization (%) in different media plates		P solubilization in broth culture after 72 hours of incubation					
					*NBRIP		†CIRP		‡PDYA-AlP	
			NBRIP	Pikovskaya	(ppm)	(%)	(ppm)	(%)	(ppm)	(%)
PSB1	10.00bc	+ve	50.56c	45.26d	104c	31.30	8.43bc	2.54	3.21c	0.97
PSB 2	9.60c	+ve	45.23d	55.23c	103c	31.00	5.67de	1.71	4.32b	1.30
PSB 3	9.00c	+ve	40.00e	40.24e	108c	32.51	7.23c	2.18	1.34e	0.40
PSB 4	5.28e	+ve	36.67f	45.65d	104c	31.30	4.32f	1.30	2.87d	0.86
PSB 5	7.32d	+ve	45.23d	50.13c	106c	31.91	10.12a	3.05	4.89ab	1.47
PSB 6	4.32f	+ve	30.22f	45.81d	109c	32.81	3.21g	0.97	3.23c	0.97
PSB 7	13.16a	+ve	57.5b	60.12b	145a	43.65	11.3a	3.40	5.43a	1.63
PSB 8	3.84fg	−ve	42.2e	45.36d	107c	32.21	6.32d	1.90	3.45c	1.04
PSB 9	4.40f	+ve	34.28f	35.93f	106c	31.91	5.64de	1.70	1.76e	0.53
PSB 10	5.44e	+ve	45.65d	50.22c	79de	23.78	6.54d	1.97	2.65d	0.80
PSB 11	9.20c	+ve	51.11c	52.16c	65f	19.57	5.21e	1.57	3.43c	1.03
PSB 12	10.00bc	+ve	55.73b	47.27d	89d	26.79	4.21f	1.27	3.28c	0.99
PSB 13	11.60b	+ve	50.32c	34.44fg	73e	21.97	4.02f	1.21	2.69d	0.81
PSB 14	5.60e	+ve	40.24e	35.38f	85d	25.59	3.41g	1.03	3.38c	1.02
PSB 15	8.16d	+ve	49.41cd	45.42d	142a	42.74	6.03d	1.82	2.76d	0.83
PSB 16	9.63c	+ve	31.11f	40.83e	112bc	33.71	4.07f	1.23	3.48c	1.05
PSB 17	12.16b	+ve	70.23a	76.03a	142a	42.74	9.34b	2.81	5.73a	1.72
PSB 18	11.36b	−ve	45.09d	44.1d	122b	36.72	5.74de	1.73	4.21b	1.27
PSB 19	10.56b	+ve	50.21c	32.5g	107c	32.21	6.24d	1.88	4.89ab	1.47
PSB 20	1.56h	+ve	23.23g	31.02g	124b	37.32	2.02h	0.61	4.1b	1.23
PSB 21	14.96a	+ve	56.65b	70.12a	144a	43.34	9.57b	2.88	5.23a	1.57

*NBRIP = National Botanical Research Institute's phosphate growth medium,

† CIRP = Christmas Island Rock Phosphate and

‡ PDYA-AlP = Potato dextrose Yeast Agar- Aluminium Phosphate respectively.

Data values are means of five replicates. Means in each column followed by the same letters are not significantly different according to Tukey's HSD at P≤0.05. Note: (+ve) for N_2_ fixing and (−) for not N_2_ fixing activities.

### Phosphate solubilization in media plates

All the selected 21 PSB isolates were able to solubilize P on NBRIP and Pikovskaya media plates, with P-solubilizing activity indicated by a clear halo zone (Plate S1). The highest P solubilizing activity in NBRIP media plate was contributed by PSB17 (70.23%), followed by PSB7 (57.5%), while the lowest activity (23.23%) was contributed by PSB20 strain (Table 5). On the other hand, on Pikovskaya media plates, the highest activity was contributed by PSB17 (76.03%), followed by PSB21 (70.12%).

### Phosphate solubilization in broth culture

The isolates from the soils were able to solubilize P from different forms of inorganic phosphate incorporated into the broth culture after 72 hours of incubation (Table 5). Comparatively, the highest P solubilization activity was found in NBRIP broth with PSB7 (43.65%), followed by PSB21 (43.34%). Most of the strains were able to solubilize P in CIRP broth, but higher P solubilization was observed in samples containing PSB1 and PSB5 compared to other isolates, and lower amounts of soluble P were observed in the PDYA-Al broth.

### Principal component analysis among 21 bacterial isolates based on biochemical properties

In order to assess the patterns of variation, PCA was done by considering seven biochemical characters. The first three principal components (PCs) explained 99% of the total variation in 21 bacterial isolates and showed 73.27, 21.63, 4.14% variations, respectively (Table 6). The PC1 and PC2were loaded with all seven characters such as IAA production, Nfb activity, P solubilization in different sources of P media and culture (NBRIP, Pikovskaya, PDYA-AlP and CIRP). All the characters either in PC1 or PC2 showed positive contribution for the differences between the bacterial isolates. The PC1 was strongly responsible for variation of the 21 bacterial isolates.

**Table 6:** Principal component analysis of seven biochemical characters and proportion of variation for each component.

Biochemical characters	aPC1	PC2	PC3
% Variation	73.27	21.63	4.14
bIAA production (mg L^−1^)	0.49485	−0.04136	0.46886
Nfb activity	0.71264	0.4016	−0.03145
P solubilization (%) on cNBRIP	0.5499	−2.0133	1.8433
P solubilization (%) on Pikovskaya media plates	0.60905	−1.5349	−2.3093
P solubilization (ppm) in NBRIP broth culture	2.4185	1.2426	0.19441
P solubilization (ppm) in dCIRP broth culture	−0.55919	0.18271	−0.0435
P solubilization (ppm) in ePDYA-AlP broth culture	−0.63265	0.33844	−0.06201

a PC = Principle component,

b IAA = Indoleacetic acid,

c NBRIP = National Botanical Research Institute's phosphate growth medium,

d CIRP = Christmas Island Rock Phosphate and

e PDYA-AlP = Potato dextrose Yeast Agar- Aluminium Phosphate respectively.

**Figure 1:**
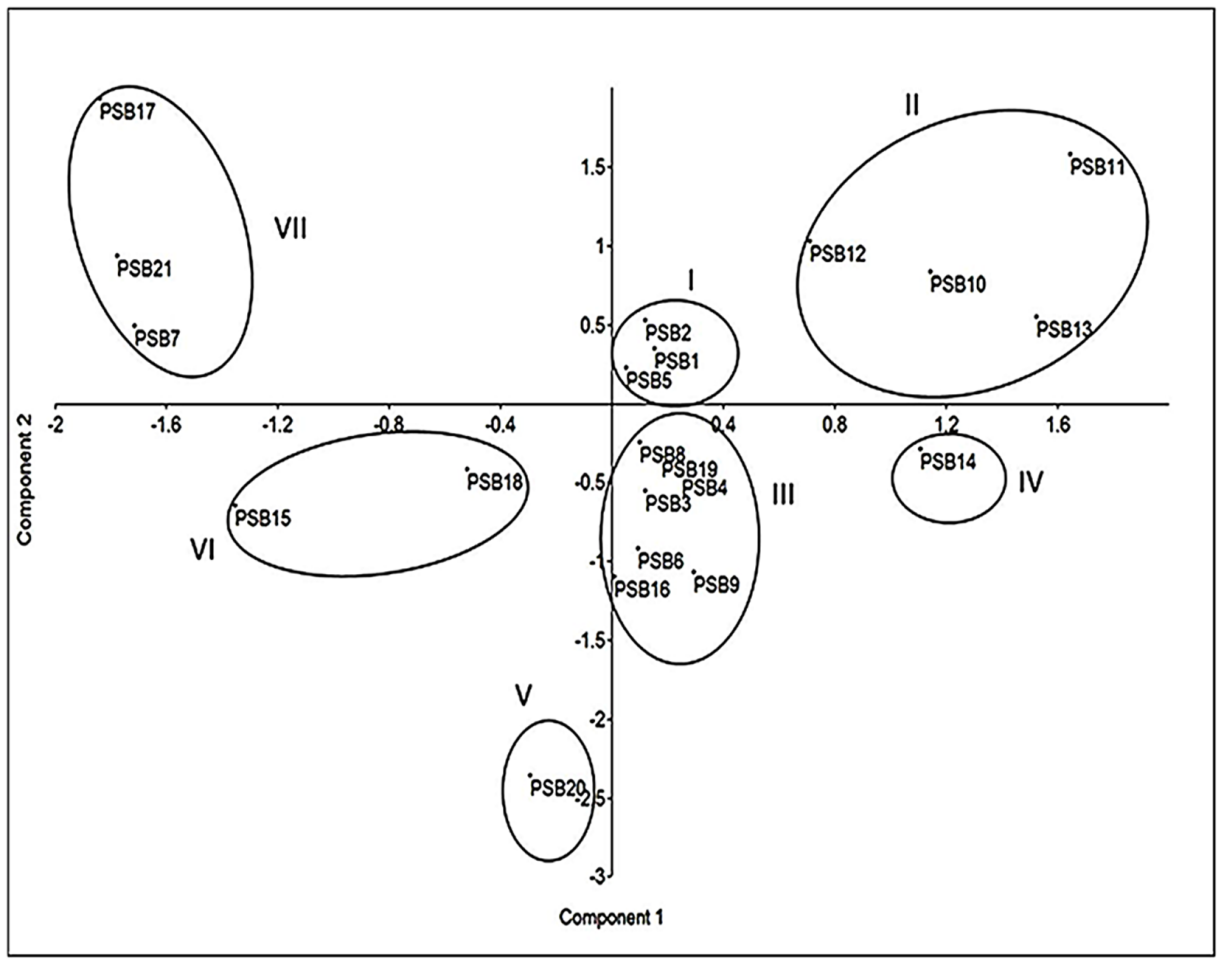
Plotting of two principal axes in principal component analysis showed the variation among 21 bacterial isolates based on seven biochemical characters using co-variance matrix.

The PCA scatter plot is shown in Fig. 1. All the bacterial isolates were grouped into seven clusters. The highest number of bacterial isolates from acid sulfate soil was in cluster III (PSB3, PSB4, PSB6, PSB8, PSB9, PSB16 and PSB19), followed by cluster II (PSB10, PSB11, PSB12 and PSB13), cluster I (PSB1, PSB2 and PSB6), cluster VII (PSB7, PSB17 and PSB21) and cluster VII (PSB15 and PSB18). Clusters IV and V each contained one strain. The results show that cluster VII with bacterial isolates PSB17, PSB21 and PSB7 was the most diverged group.

### Identification of the potential strains

**Figure 2:**
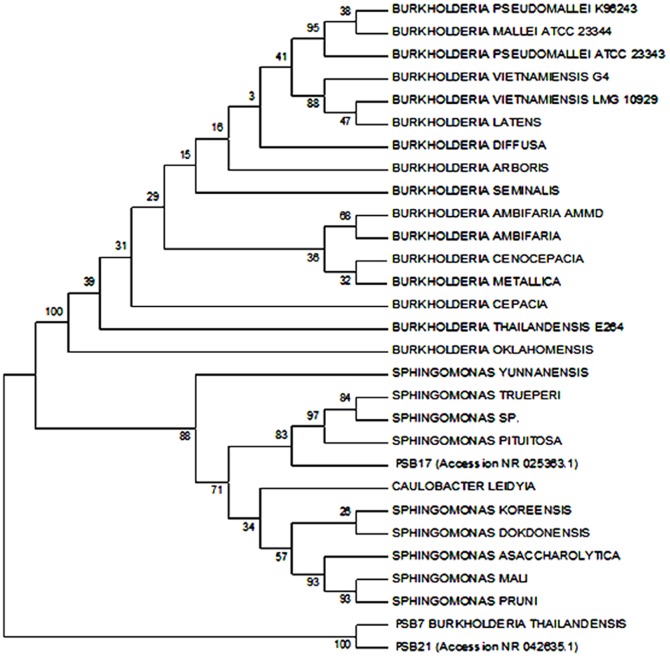
Phylogenetic tree with bootstrap values. Tree constructed using Neighbor-Joining (NJ) method. PSB7 accession NR 074312.1, PSB17 accession NR025363.1, and PSB21 accession NR 042635.1.

**Figure 3:**
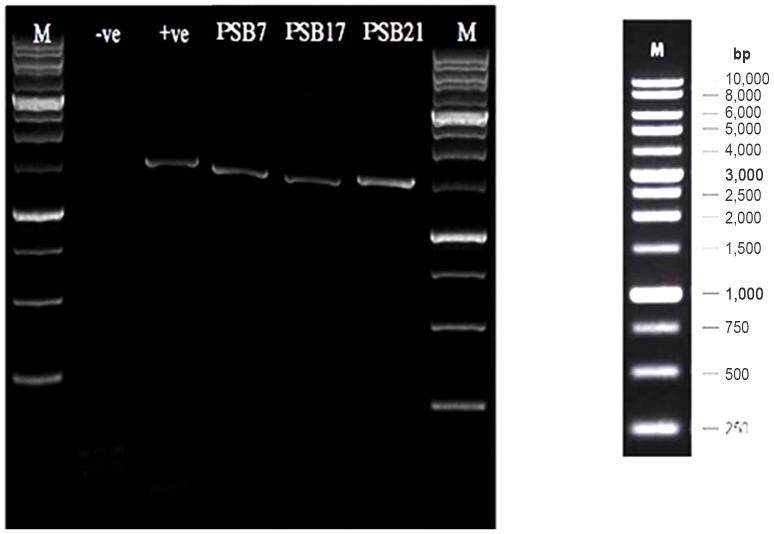
SDS-PAGE of the PSB7 (*Burkholderia thailandensis*), PSB17 (*Sphingomonas pituitosa*) and PSB21 (*Burkholderia seminalis*). (M: DNA ladder; −ve: negative control; +ve: positive control).

Molecular analysis by the 16S rDNA identification technique was adopted in this study. These excellent markers for the clarification of bacterial phylogeny are ribosomal ribonucleic acids. In this study, we used gene sequences from the β-subclass of Proteobacteria to determine the phylogenetic relationships among the tested isolates. The neighbor-joining tree was subjected to the numerical re-sampling by bootstrapping, and the resulting bootstrap values were observed at the tree branch nodes. Each value represents the number of times (out of 1000 replicates) that the represented groupings occurred in the re-samplings. The consensus tree showed 98–100% confidence levels between 3 potential isolates from the β-subclass of Proteobacteria PSB7 [*Burkholderia thailandensis*] and PSB21 [*Burkholderia seminalis*], whereas PSB17 [*Sphingomonas pituitosa*] had a 100% confidence level (Fig. 2 & 3).

All of the sequences have higher than 98% identity with the queried sequence. Two PSB7 and PSB21 sequences were identified as *Burkholderia* spp. with accession numbers NR 074312.1 and NR 042635.1, respectively, while one belonged to *Sphingomonas* sp. (NR 25363.1).

### The efficacy of PSB strains to reduce Al toxicity

It was observed that high Al concentration severely affected the growth of the rice seedlings. Plant height and dry biomass significantly decreased with increased Al concentrations (Table 7). In comparison, the bacterial inoculated plants were less affected by Al toxicity. High plant height (18 cm) and dry biomass (0.76 g) were observed in the *Burkholderia seminalis* inoculated treatments at 0 µM Al (Table 7). The PSB associated with the plant roots produced organic acids that provided P and chelated the Al toxicity. As shown by the SEM and TEM micrographs (Plate S2), the bacteria existed in association with the rice seedlings.

**Table 7:** Effects of Al and PSB inoculation on the growth of rice seedlings.

Treatments	Plant height (cm)			Dry weight (g)			Root length (cm)			Root surface area (cm^2^)			Root volume (cm^3^)		
	---------------------------------------------------------- Al conc. (µM) ----------------------------------------------------------------														
	0	50	100	0	50	100	0	50	100	0	50	100	0	50	100
Control	14.5c	13.3c	11.6c	0.61c	0.59c	0.58c	24.31c	23.69c	18.99d	42.63d	28.29d	25.47c	3.51c	2.62d	1.03c
*Burkholderia thailandensis*	17.7b	15.6b	14.1a	0.71b	0.69a	0.67a	57.69b	36.32b	33.78c	69.08c	58.43a	42.36a	4.80b	4.19b	3.42a
*Burkholderia seminalis*	18a	16.4a	14.1a	0.76a	0.69a	0.65b	58.02a	47.28a	36.85b	74.12a	53.02b	43.40a	6.88a	3.76c	2.03b
*Sphingomonas pituitosa*	17b	15b	13b	0.71b	0.66b	0.65b	58.51a	49.66a	38.01a	72.59b	45.45c	38.52b	6.90a	5.18a	3.81a

Data values are means of five replicates. Means within the same column followed by the same letters are not significantly different at *P*<0.05.

### The morphology of the PSB-inoculated and untreated rice roots

This study found that root length, root surface area and volume varied with the Al concentration and bacterial inoculation. Presence of Al affected rice root development, especially at high concentrations (Table 7). Generally, greater root length, surface area and volume were found in inoculated compared to non-inoculated rice plants. Significantly greatest root length (58.51 cm) was observed at 0 µM Al by PSB21 inoculated rice plant compared those at high Al concentration. The greatest root surface area of 74.12 cm^2^ was recorded in PSB17 inoculated rice at 0 µM Al, while the highest root volume was observed in the *Sphingomonas pituitosa* and *Burkholderia seminalis* (6.9 and 6.88 cm^3^, respectively) inoculated rice plants at the same Al concentration.

### Effects of Al and PSB inoculation on the release of organic acids

It was observed that organic acids released by the rice roots varied with the Al concentration and bacterial inoculation (Table 8). Plants with highest Al concentration was found to secrete the highest amounts of organic acids. The release of organic acids was affected by the Al concentration. Higher amounts of organic acids were released by PSB-inoculated plants compared to the non-inoculated rice seedlings. Among the organic acids released, higher amounts of oxalic and citric acids were observed at 100 µM Al compared to malic acid, while the amount of malic acid was found to be low, particularly at 0 and 50 µM Al concentration.

**Table 8:** Effects of Al and PSB inoculation on the release of organic acids.

Treatments	Oxalic acid (µM)			Citric acid (µM)			Malic acid (µM)		
	--------------------------------- Al conc. (µM) ----------------------------------								
	0	50	100	0	50	100	0	50	100
Control	72c	82d	94d	13d	40d	52d	3d	4d	55d
*Burkholderia thailandensis*	78c	131a	265 a	45b	196a	257b	40a	121a	336a
*Burkholderia seminalis*	98a	123b	114 c	146a	170b	268a	7b	86b	315b
*Sphingomonas pituitosa*	95ab	94c	185 b	38c	137c	241c	5c	54c	151c

Data values are means of five replicates. Means within the same column followed by the same letters are not significantly different at *P*<0.05.

### Effect of Al on the population of PSB strains with or without rice seedlings

**Figure 4:**
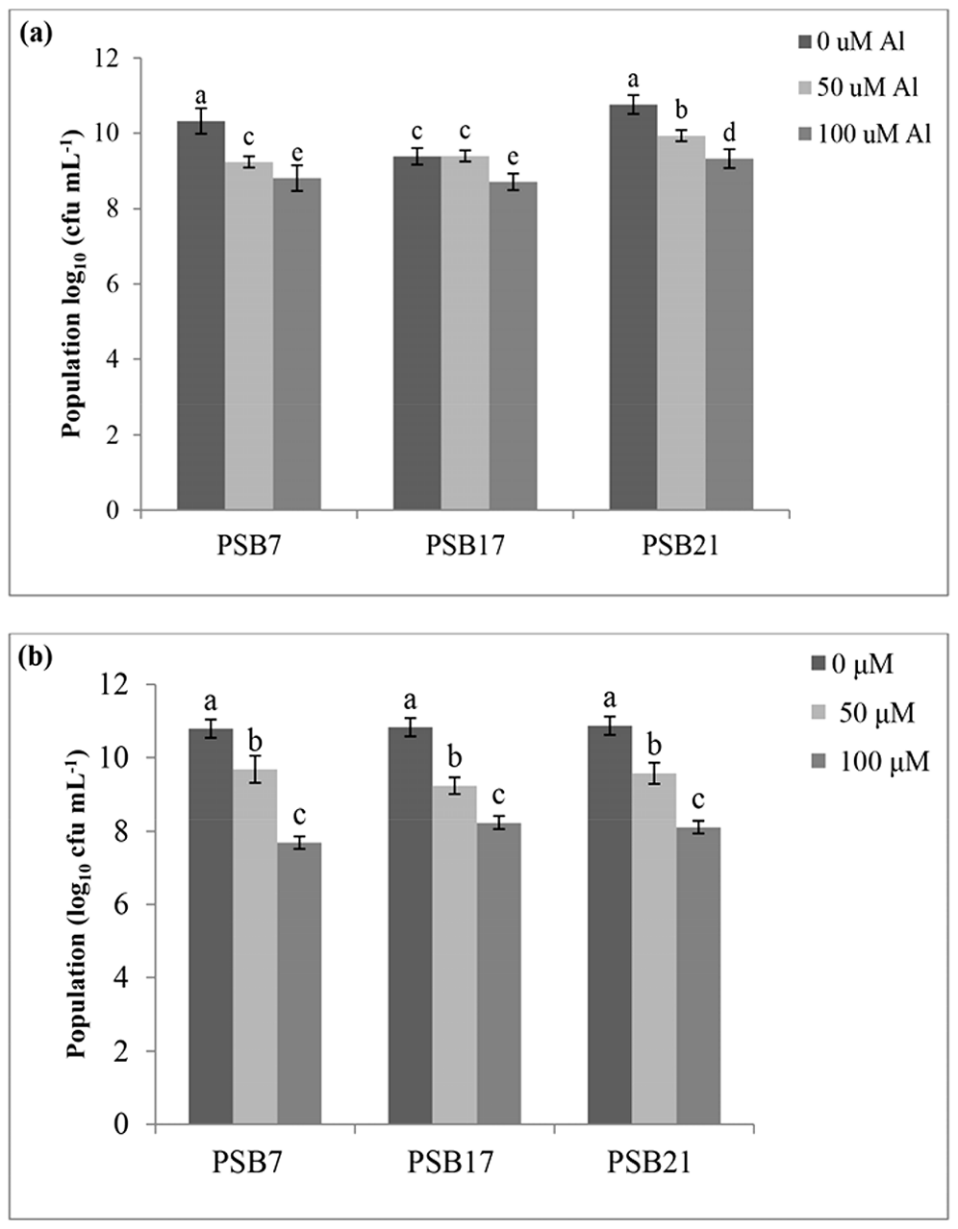
Effect of different Al concentrations on the PSB population (a) without plant, (b) with plant system.

It was found that the population of PSB was affected by the Al concentrations in both of the plant and without plant systems. It seemed that a higher Al concentration lowered the population of PSB (Fig. 4a &b). Hence, at 0 µM Al concentration, higher PSB population was observed compared to that observed at higher Al concentrations. Among the inoculated PSB strains, the highest population was recorded by PSB21 at all the Al concentrations without plant system, while in plant system all strains were showing the same response. The population decreased with the increasing Al concentration, and this trend was observed for all the PSB strains in both culture systems. It was clear from the current study that some bacteria strains survived under the condition of low pH and high Al concentration; these strains have the potential to be used for rice cultivated on acid sulfate soil containing high Al concentration.

### Effect of PSB inoculation on nutrient solution pH at different Al concentration

**Figure 5:**
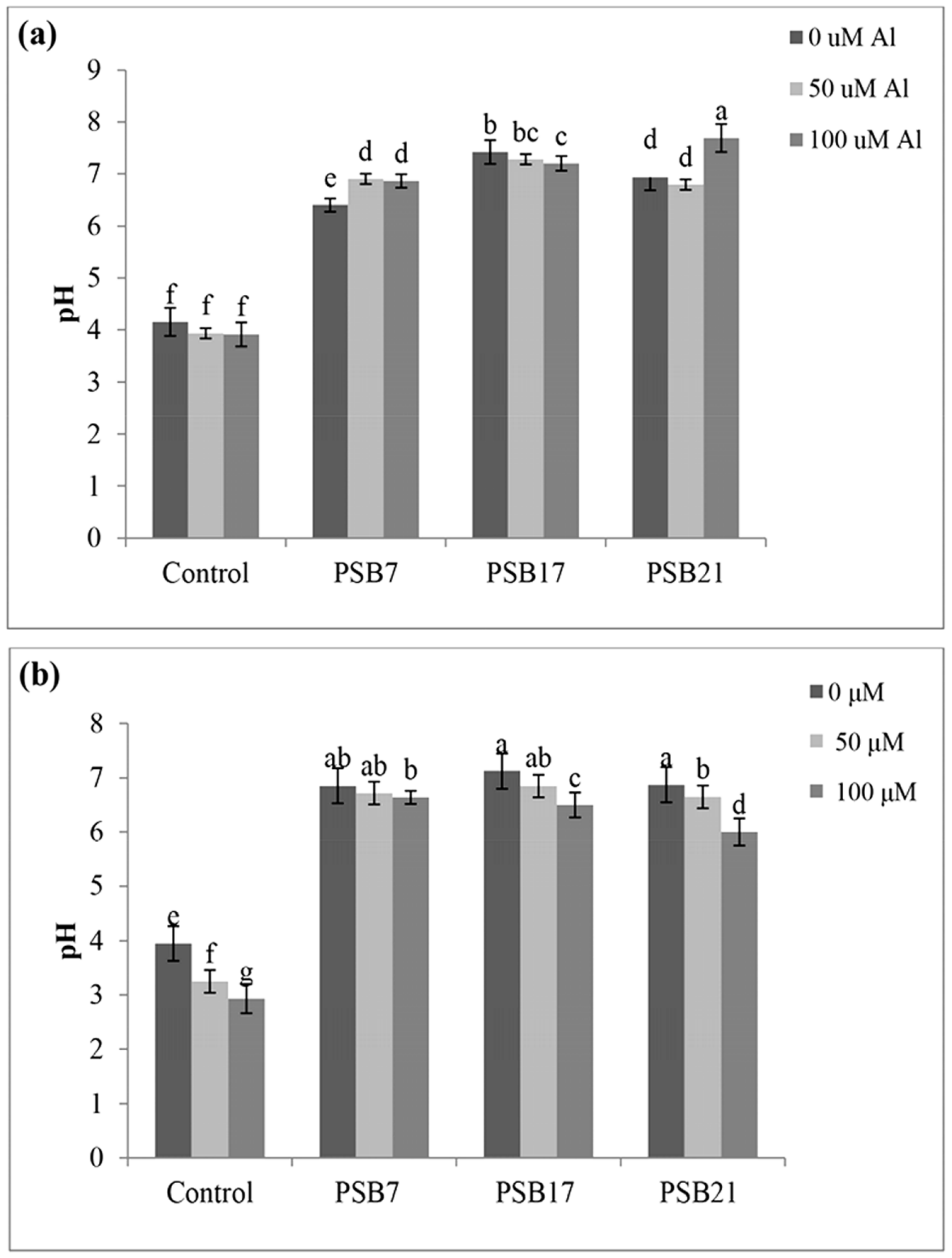
Effect of PSB inoculation on solution pH at different Al concentration a) without plant, b) with plant.

**Figure 6:**
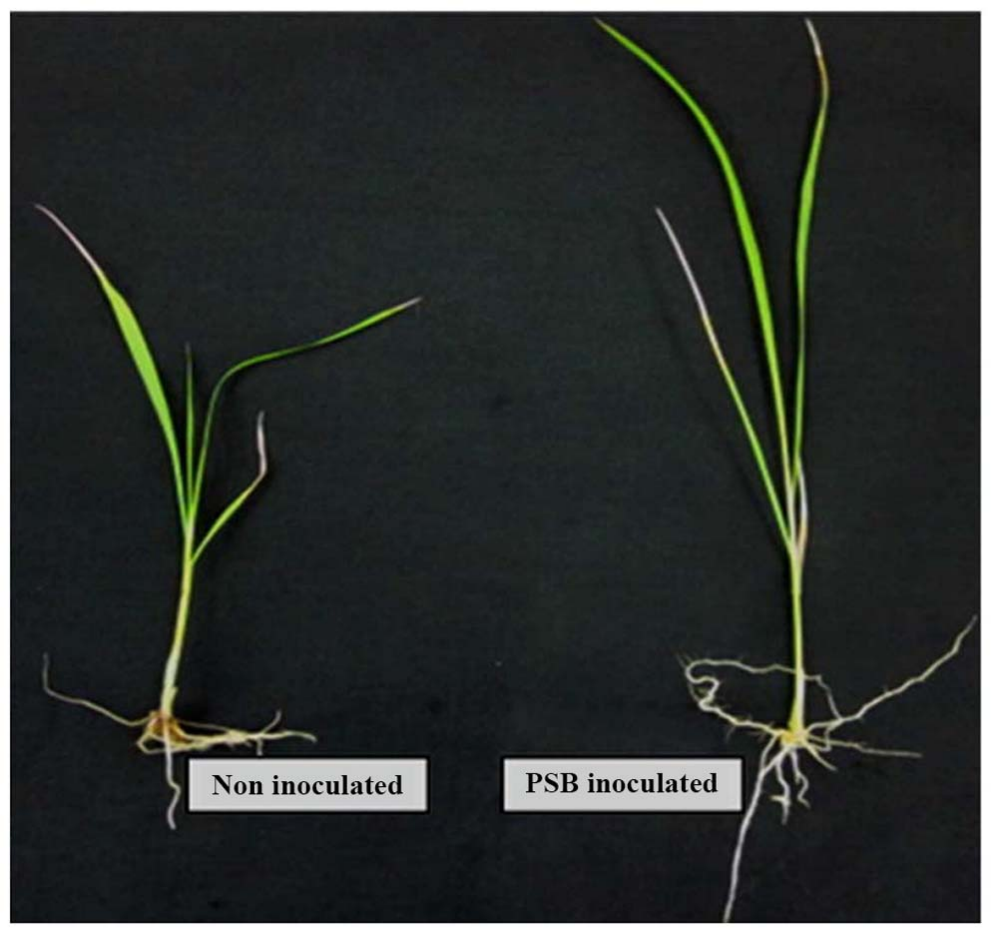
Effect of Al concentration (100 µM) at pH 4.0 on rice seedlings.

There was a clear effect of PSB inoculation found in the nutrient solution pH at different Al concentrations in both of the plant and without plant systems (Fig. 5a &b). The initial solution pH was 4.0 and after 24 h of inoculation, the pH increased to 7.00 for all PSB inoculated treatments (with and without plant system). However, for the non-inoculated treatment, it remained almost the same as the initial pH (3 to 4.0) in both of the conditions regardless of Al concentrations. Without plant, the highest pH of 7.69 was obtained for the 100 µM Al treatment with PSB 21(Fig. 5a). For the inoculated plant, the solution pH was a bit higher in 0 µM Al compared to the other treatments (Fig. 5b). The lowest solution pH of 2.80 was found in the non-inoculated control at 100 µM of Al concentration (with rice seedling). Rice seedlings were affected severely by the low pH. However, PSB inoculation increased the pH of solution that resulted in enhanced growth of rice seedlings (Fig. 6).

## Discussion

Acid sulfate soils are dominated by sulfur or oxidizing-reducing bacteria [28]. These soils may also contain some beneficial microorganisms that help improve rice growth. In this study, it was found that the pH of the acid sulfate soils was very low and Al concentration was very high, especially at the depth below 50 cm, proving that these were true acid sulfate soils [29]. Although the soils were very acidic there were still some microorganisms living in them. These microorganisms (bacteria, actinomycetes and fungus) can be potentially beneficial for rice production. It was found that the population of the bacteria changed from place to place and varied according to the native vegetation. Furthermore, the bacterial population was found to be higher at the rhizosphere compared to the non-rhizosphere, indicating bacterial synergism with plant roots [11].

A total of 21 PSB were isolated from the acid sulfate soils in the present study. These PSB isolates have the potential to be used in rice production. Higher PSB populations were observed in the calcium phosphate (NBRIP) medium compared to other P media sources. In this medium, the PSB population was not affected by pH. Panhwar *et al.* [13] reported significantly higher PSB population observed in tricalcium phosphate medium. Furthermore, it was reported that most of the strains after an incubation period formed pellicles in N free semisolid-malate media, pointing to their N_2-_fixing abilities [30].

The isolated strains have the capability to produce growth hormones (IAA) that could help to the plants to enhance their root and shoot growth. This shows the potential of these bacteria for use in crop production [31]. It is known that rhizosphere microorganisms mediate many soil processes, such as decomposition, nutrient mineralization and nitrogen fixation [32]. Other researchers have reported that bacteria in rice fields have the potential to produce IAA and are able to fix N [11]–[33]. In the current study, it was found that the isolated strains were able to solubilize phosphate in NBRIP and Pikovskaya media, with P-solubilizing activity indicated by clear halo zone around colonies [34]–[35]. A similar trend was observed for liquid broth culture using calcium phosphate (NBRIP) and rock phosphate (CIRP). The highest P solubilization occurred in NBRIP broth, while the lowest was in the CIRP and PDYA-AlP broth cultures. This is similar to the findings of Chakraborty *et al.* [36] who reported that isolated PSB strains solubilized P from calcium phosphate to a greater amount than rock phosphate, aluminum phosphate and iron phosphate.

The bacterial population was significantly affected by the different forms of inorganic phosphate incorporated into the broth culture. NBRIP and CIRP broths were found to have the highest bacterial population, while lower bacterial growth was found in the PDYA-AlP broth after 72 hours of inoculation. The low content of solubilized P in the PDYA-A broth may be due to its insolubility although the isolates had the ability to solubilise it. Al in the soils may fix P, even further reducing solubilization. It has been established that phosphate rock has a lower content of soluble P compared to calcium phosphate [37].

The three principal components showed 99% variation of the total variation in 21 bacterial isolates. Similarly, Naher *et al.* [38] found distinct variations in different soil bacterial isolates using principal component analysis. Based on the production of indoleacetic acid and phosphate solubilizing activities, three isolates PSB7, PSB21 and PSB21 were found to have great potential. This noteworthy finding is supported by the PCA scatter plot based on the biochemical properties and these three bacterial isolates which were grouped distantly from the other strains.

The assessment of the bacterial 16S rRNA gene sequence has emerged as a preferred genetic technique as it can better identify weakly described, rarely isolated, or phenotypically aberrant strains [39]–[40]. In our study, we employed a molecular phylogenetic approach based on 16S rRNA sequences to identify pure potential isolates. Three potential isolates, namely PSB7 (*Burkholderia thailandensis*), PSB21 (*Burkholderia seminalis*), and PSB17 (*Sphingomonas pituitosa*) were identified.

The present isolates demonstrated beneficial traits like N_2_ fixation, phosphate solubilization and phytohormones production (IAA). Several *Burkholderia* spp. (*Burkholderia unamae*, *Burkholderia xenovorans*, *Burkholderia silvatlantica*, *Burkholderia tropica*, *Burkholderia tuberum*, *Burkholderia phymatum*, *Burkholderia mimosarum* and *Burkholderia nodosa*) have been known to fix N_2_ [41] and are commonly find in rice, maize, sugar cane, sorghum, coffee and tomato [42]. The *Sphingomonas* sp. has beneficial attributes like N_2_ fixing [43] and high polysaccharide production. These species are able to synthesize the bacterial exopolysaccharide gellan and related polymers and were shown to possess constitutive gellanase activity [44]. Exopolysaccharides have been known to perform a major role in providing protection to the cell as a boundary layer [45], as well as by chelating heavy metals due to the presence of several active functional groups [46].

The isolated PSB were able to grow well under low pH conditions. This might be due to the deposition of lipopolysaccharide in bacterial cultures at a pH below 4.5. The possibility of lipopolysaccharide incorporation into the cell wall is episodic at acidic pH that would argue against the involvement of these polysaccharides in the acid pH toterant bacteria (*R. tropici* UMR1899) as other outer-membrane components may confer greater pH tolerance to the strain [47]. Exopolysaccharides were partially distinguished by their FTIR spectra indicating the presence of carboxylic acid group and H^−^ bonded group that might have been the reason for the increase of pH in broth culture [44]. However, in the present study, increase in the pH of the nutrient solution from 4 to 7 (both plant and without plant system) might be the result of bacterial exopolysaccharide production. An increase in the pH of bacterial culture medium was the main factor that governs cell growth and exopolysaccharides production [48]. Moreover, bacteria produce polysaccharides in the presence of carbon source and when the pH of the broth is increased from 5.0 to 7.0 [49].

Al toxicity is one of the major constraints to rice root development. In the present study, PSB-inoculated rice seedlings showed better root growth compared to non-inoculated seedlings. The pH of the solution changed from acidic to neutral, providing a favourable environment for plant growth. The better root growth was also promoted by the release of organic acids produced by PSB that chelated Al, rendering it inactive [4]. It is known that rice roots also secrete organic acids (citric, oxalic, and malic acids) and the secretion of these organic acids is localized to the root apex [50]. The inoculated rice seedlings without the presence of Al produced higher plant biomass. This might have been due to the production of phytohormone by the bacteria (such as IAA) that stimulated rice plant growth.

In the present study, multiple reasons might have contributed to a reasonable seedling growth under high Al and low pH conditions. First, the strains were able to produce some polysaccharides that increased the solution pH. Moreover, the strains produced organic acids that helped chelate Al in the solution and consequently reduced Al toxicity. The organic acids performed two main important functions: 1) to detoxify Al; and 2) to make P available [51]. There has been evidence of aluminum resistance in some plant species that has been ascribed to organic acid exudation from roots and by microorganisms that have the ability to chelate Al [52]. However, the most effective organic acids to chelate Al, in decreasing order, are citric, oxalic and tartaric acids [53].

In this study, it is proven that these isolates have the potential to reduce Al toxicity, fix nitrogen, solubilize phosphate, increase soil pH and produce phytohormone. Therefore, these three bacteria strains can be exploited to enhance the productivity of rice planted on acid sulfate soils either in Malaysia or other part of the tropics.

## Conclusions

Rice growing on acid sulfate soils suffers from Al^3+^ toxicity and H^+^ which affects its growth and eventually the yield. To some extent, rice with the help of PSB is able to reduce Al^3+^ by excreting organic acids (oxalic, citric and malic acids) via its roots at the appropriate time, and these acids are able to detoxify Al^3+^ via a chelation process. The growth of rice can be improved further with the help of PSB. In this study, three locally isolated bacteria; *Burkholderia thailandensis* (PSB7), *Sphingomonas pituitosa* (PSB17) and *Burkholderia seminalis* (PSB21) of these essential microbes were found to exist under adverse conditions (low pH and high Al concentration). These PSB not only helped to solubilize P in the soils, but their activities resulted in increased pH due to the release of exo-polysaccharides. If the water pH increased above 5, Al would have precipitated as inert Al-hydroxides, making it unavailable to the growing rice in the field. These PSB could also produce growth enhancement phytohormones such as IAA. The potential of these PSB should be exploited further for the sustainable management of acid sulfate soils, especially for rice production. For instance, they can be used as bio-fertilizers to improve the fertility of acid sulfate soils.

## Supporting Information

Plate S1 **P solubilization on media plate.**
(DOCX)

Plate S2 **a) SEM micrograph and b) TEM micrograph of the PSB living on the surface and inside the rice root.**
(DOCX)
