# Correction: Biochemical and Molecular Characterization of Potential Phosphate-Solubilizing Bacteria in Acid Sulfate Soils and Their Beneficial Effects on Rice Growth

**DOI:** 10.1371/journal.pone.0116035

**Published:** 2014-12-16

**Authors:** The PLOS ONE Staff

The third author’s name is displayed incorrectly. The correct display is: Jusop Shamshuddin. The correct citation is: Panhwar QA, Naher UA, Shamshuddin J, Othman R, Latif MA, et al. (2014) Biochemical and Molecular Characterization of Potential Phosphate-Solubilizing Bacteria in Acid Sulfate Soils and Their Beneficial Effects on Rice Growth. PLoS ONE 9(10): e97241. doi:10.1371/journal.pone.0097241
