# Supplementary figures and images for: Biochemical and Molecular Characterization of Potential Phosphate-Solubilizing Bacteria in Acid Sulfate Soils and Their Beneficial Effects on Rice Growth
Source: PLoS One. 2014 Oct 6;9(10):e97241. doi: 10.1371/journal.pone.0097241 (PMC4186749; doi:10.1371/journal.pone.0097241)

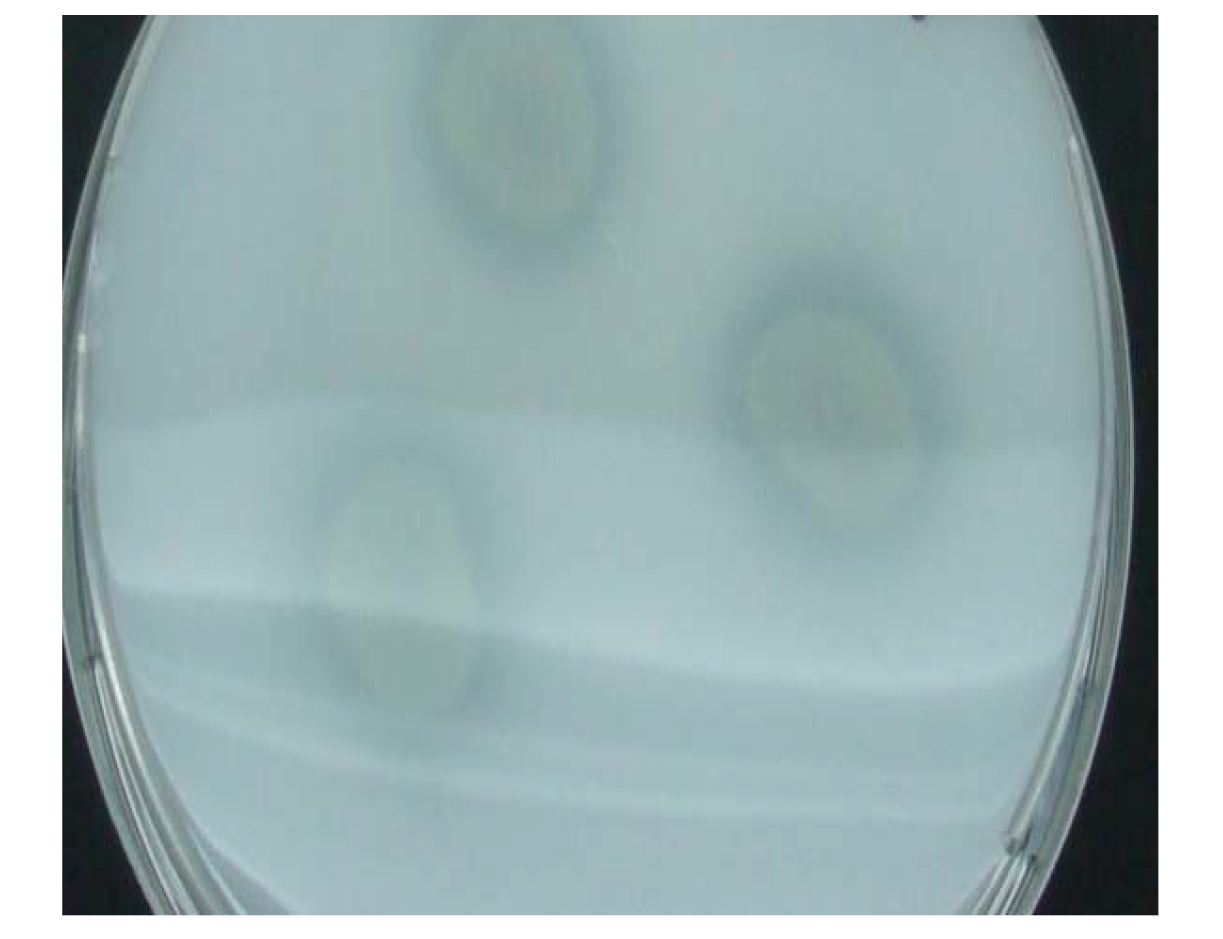


**Plate 1. P solubilization on media plate**

Supplement: Plate S1 — P solubilization on media plate. (DOCX) [file pone.0097241.s001.docx]
