# Supplementary material for: Biochemical and Molecular Characterization of Potential Phosphate-Solubilizing Bacteria in Acid Sulfate Soils and Their Beneficial Effects on Rice Growth
Source: PLoS One. 2014 Oct 6;9(10):e97241. doi: 10.1371/journal.pone.0097241 (PMC4186749; doi:10.1371/journal.pone.0097241)
Supplement: Plate S2 — a) SEM micrograph and b) TEM micrograph of the PSB living on the surface and inside the rice root. (DOCX) [file pone.0097241.s002.docx]

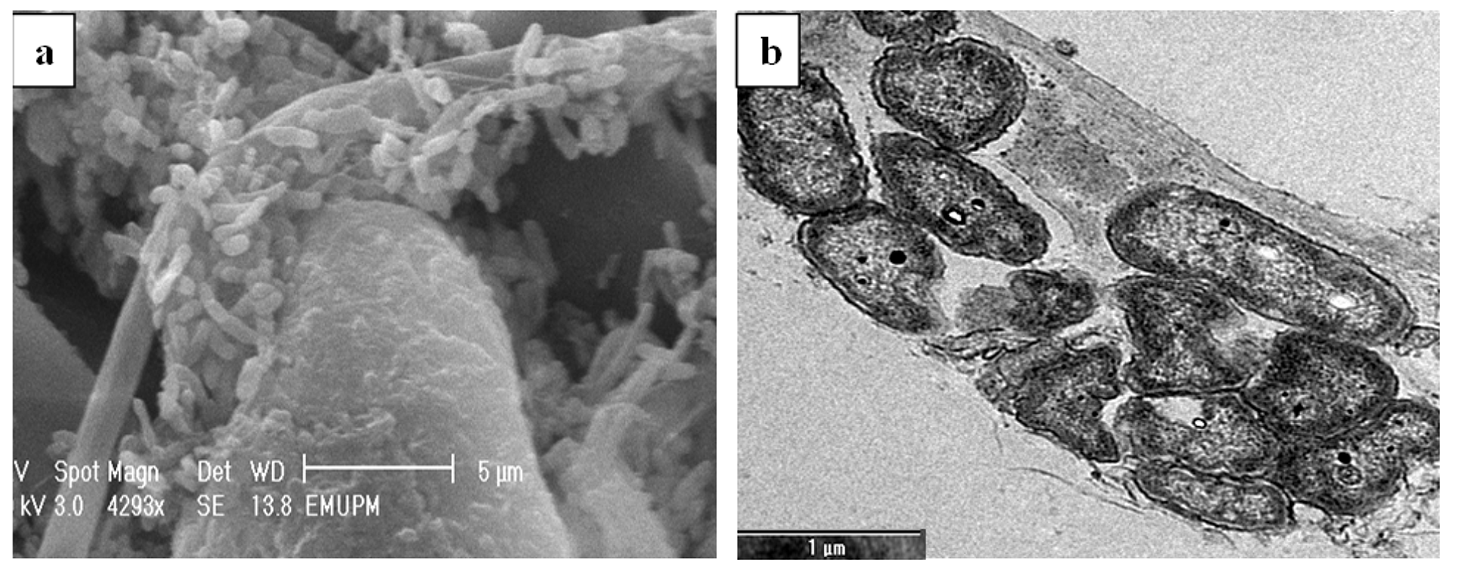


**Plate 2. a): SEM micrograph and b) TEM micrograph of the PSB living on the surface and inside the rice root**
